# Is there excess mortality in women screened with mammography: a meta-analysis of non-breast cancer mortality

**DOI:** 10.1186/1745-6215-14-368

**Published:** 2013-11-05

**Authors:** Sylvie Erpeldinger, Laure Fayolle, Rémy Boussageon, Marie Flori, Xavier Lainé, Alain Moreau, François Gueyffier

**Affiliations:** 1 Department of General Medicine, Université Claude Bernard Lyon1, 69000, Lyon, France; 2 Department of Clinical Pharmacology, Hospices Civils de Lyon, 69000, Lyon, France; 3 Service de Pharmacologie Clinique, Université Claude Bernard Lyon 1, 69000, Lyon, France

**Keywords:** Breast cancer, France, Mammography, Meta-analysis, Mortality, Screening

## Abstract

**Background:**

The objective of our meta-analysis and systematic review was to analyze non-breast cancer mortality in women screened with mammography versus non-screened women to determine whether there is excess mortality caused by screening.

**Methods:**

We searched PubMed and the Web of Science up to 30 November 2010. We included randomized controlled trials with non-breast cancer mortality as the main endpoint. Two authors independently assessed trial quality and extracted data.

**Results:**

There was no significant difference between groups at 13-year follow-up (odds ratio = 1.00 (95% CI 0.98 to 1.03) with average heterogeneity I^2^ = 61%) regardless of the age and the methodological quality of the included studies. The meta-analysis did not reveal excess non-breast cancer mortality caused by screening. If screening does have an effect on excess mortality, it is possible to provide an estimate of its maximum value through the upper confidence interval in good-quality methodological studies: up to 3% in the screened women group (12 deaths per 100,000 women).

**Conclusions:**

The all-cause death rate was not significantly reduced by screening when compared to the rate observed in unscreened women. However, mammography screening does not seem to induce excess mortality. These findings improve information given to patients. Finding more comprehensive data is now going to be difficult given the complexity of the studies. Individual modeling should be used because the studies fail to include all the aspects of a complex situation. The risk/benefit analysis of screening needs to be regularly and independently reassessed.

## Background

Breast cancer is the most common cancer in women worldwide and accounts for 16% of all female cancers. In 2004, nearly 519,000 women died of breast cancer [1]. In France, breast-cancer mortality is the leading cause of cancer death in women with 11,886 deaths in 2012 [2]. Although a decrease has been observed in the standardized mortality ratio for breast cancer patients worldwide (6.8/100,000 women from 2000 to 2008) [2], the incidence of breast cancer nearly doubled in 25 years, from 56.8/100,000 women in 1980 to 101.5/100,000 women in 2005 (standardized incidence rates (world population)) [3].

Developed countries set up mass screening of breast cancer with mammography in order to reduce breast cancer mortality. In France, mass screening without advance payment was implemented throughout the country in 2004 for all women between the age of 50 and 74 years (except for at-risk women) [4,5]. It is recommended that women undergo mammography screening (two-view and double read) every 2 years [6]. The participation rate for mass screening is low (52% in 2010). Apart from mass screening, individual screening continues to be used.

Many meta-analyses related to mass screening have been published and showed that mammography screening is efficient in reducing breast cancer mortality [7-11]. A Cochrane meta-analysis, published in 2001 and updated in 2009 and 2011, showed a reduction in breast cancer mortality in women screened with mammography (relative risk (RR) 0.75, 95% CI 0.67 to 0.83) but without a reduction in overall mortality (RR 0.99, 95% CI 0.95 to 1.03). The authors concluded that out of 2,000 women screened for 10 years, 1 had prolonged life expectancy, and 10 received unnecessary treatment because they were healthy and would not have been diagnosed without screening. In addition, more than 200 women suffered from serious psychological distress for various months due to false-positive results [12,13].

This meta-analysis raises questions about the risk/benefit analysis of breast cancer screening with mammography. It emphasizes the idea of excess morbidity and mortality caused by screening but not related to breast cancer and especially mortality caused by overdiagnosis and overtreatment. The purpose of the current study was to determine if there is excess mortality caused by mammography screening. A meta-analysis of randomized controlled trials (RCTs) was performed. The endpoint was non-breast cancer mortality in the screened group versus control group. The blinded assessment of outcomes warrants the equal distribution of bias between the compared groups of each study.

## Methods

The study included RCTs involving women over 39 years of age with no history of breast cancer and who underwent mammography screening (study group) versus those who did not (control group). The main endpoint was non-breast cancer mortality at 13-year follow-up and for all ages (with age subgroup analyses, in other words under or over 50 years old), depending on the methodological quality of the included studies. The 13-year follow-up was calculated from randomization.

### Search strategy

PubMed and the Web of Science were searched up to 30 November 2010. MeSH keywords [breast neoplasms, mammography and mass screening] were combined with other keywords [breast cancer, mammograph*, screen*] with AND, except for synonyms, which were combined with OR. The literature search was restricted to randomized controlled trials and meta-analyses.

### Assessment of potential bias and data collection

Two authors (LF and SE) independently assessed trial quality and extracted data. They analyzed the internal validity of studies by answering the questions from the French Cochrane Centre’s tutorial designed for assessing studies, based on the PRISMA statement [14]. These articles were then rated according to methodological quality: good, moderate, and low. They were assessed according to randomization quality, classification method for the cause of death, post-randomization exclusions, contamination bias of the control group, and compliance bias of the study group.

Data were collected from primary studies and compared with Cochrane Library data. When there was missing data, we used those of the Cochrane Library. If there was a difference between primary study data and Cochrane Library data, the first were used. Death rates were standardized (CI calculated for 100,000 people/year) in order to assess non-breast cancer mortality (overall mortality minus breast cancer mortality).

### Statistical analyses

We used RevMan 5® software to analyze data (Review Manager (RevMan) [Computer program]. Version 5.2. Copenhagen: The Nordic Cochrane Centre, The Cochrane Collaboration, 2012). The quantitative analysis of events was based on the intention-to-treat principle. Odds ratios (ORs) and a fixed-effect model were used to combine events between studies. Heterogeneity was calculated with the I^2^ test. The alpha value for the included events was considered statistically significant when < 0.05. The following formula was used to estimate the annual rate (that is, the number of deaths averted or caused by screening for 100,000 people/year): annual rate = total number of events / (all women × duration of follow-up). To estimate the number of averted deaths (positive results) or caused deaths (negative results), we multiplied the annual rate by the relative risk reduction, which was calculated by subtracting 1 from the odds ratio. The same transformation was applied to the endpoints of the CI.

## Results

**Figure 1:**
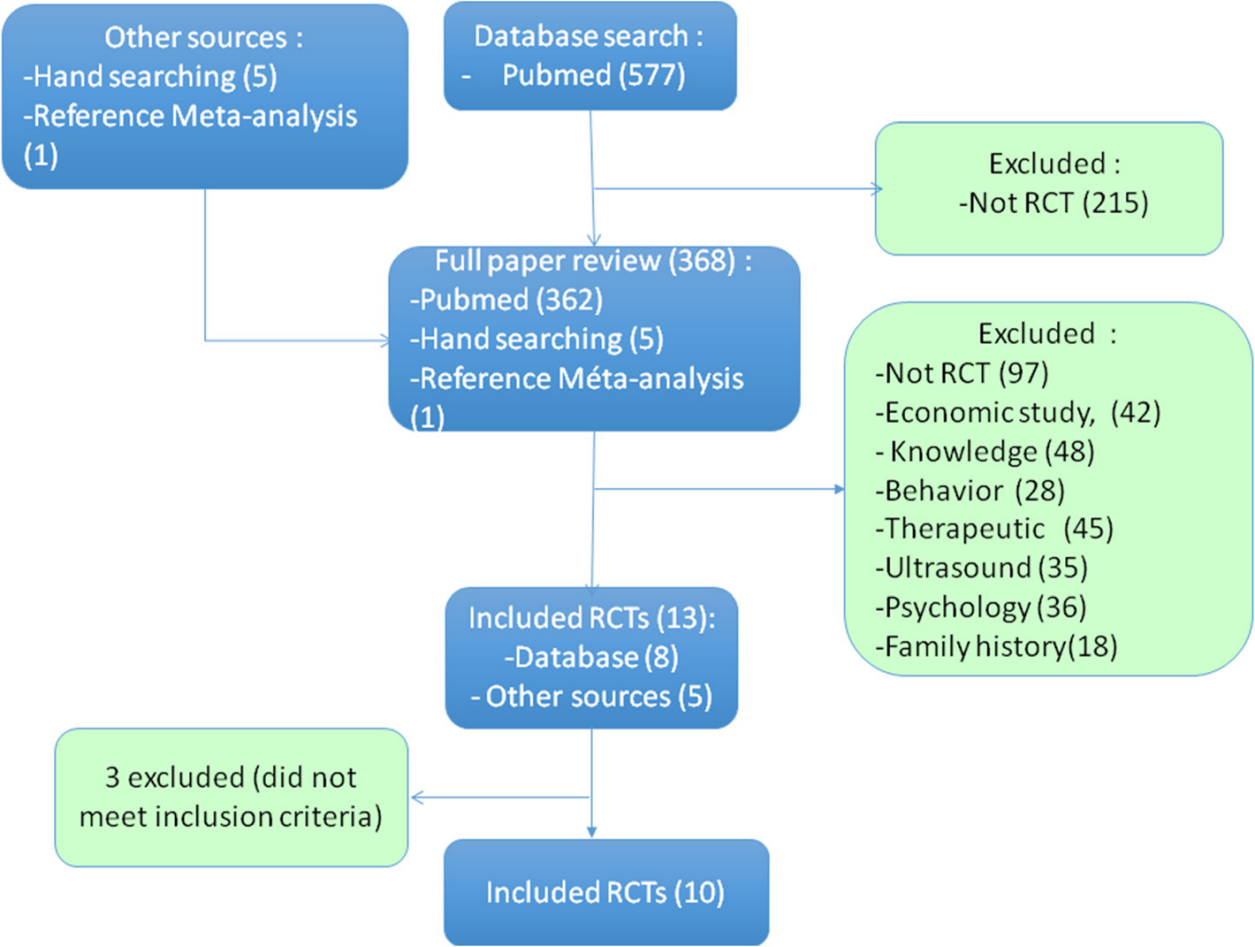
**Flow diagram of included studies.** RCT, randomized controlled trial.

A total of 577 articles were selected based on our inclusion criteria (Figure 1). Among these, 13 studies were included once the titles, abstracts, and full articles were analyzed. Three of them were excluded because they did not comply with our inclusion criteria: Berglund 2000 [15], a comparative study of cardiovascular morbidity and mortality that involved various types of examination (including mammography) in a population of men and women; Singapore 1994 [16], a comparative study of 166,600 women aged 50 to 64 with screening prevalence as an endpoint; and India 2010 [17], a controlled randomized study of 151,538 women using a clinical examination of breasts without mammography.

Four of the included studies involved clinical examination combined with mammography screening (Canada 1 & 2, New York, and Edinburgh). Two authors (LF and SE) independently analyzed data with the Cochrane Collaboration’s tool for assessing risk of bias of included studies. After pooling their results, they classified the studies according to methodological quality. The methodological quality of studies was rated good, moderate, or low. Out of the 10 included studies: 4 were considered of good methodological quality (UK Age Trial [18,19], Canada 1 & 2 [20-23], Malmo 1 [7,8]); 5 were considered of moderate methodological quality (Goteborg [24,25], Malmo 2 [26,27], Stockholm [28], Ostergotland & Kopparberg [29-31]), New York [32-34]; and 1 was considered of low methodological quality (Edinburgh) [35] (Table 1). Edinburgh’s findings were excluded from the analyses because of its low methodological quality.

**Table 1:** Description of included studies

**Clinical trial**	**Country-City**	**Year**	**Patient age**	**Type of examination**
			**(years)**	
UK Age Trial [17,18]	England, Wales, Scotland	1991	39-41	Mammography: 2 views, and then 1 view every 12 months
Goteborg [7,8,19,20]	Goteborg, Sweden	1982	39-59	Mammography: 2 views, and then 1 view every 18 months, 4 to 5 screening rounds
Malmo [7,8,21,22]	Malmo, Sweden	MMST 1: 1976	MMST 1: 44–68	Mammography: 2 views (first 2 rounds) and then 1 view every 18 to 24 months, 5 screening rounds
		MMST 2: 1978	MMST 2: 45-50	
Stockholm [8,23]	Stockholm, Sweden	1981	40-64	Mammography: 1 view every 24 months, 2 screening rounds
Canada [24-27]	15 centers, Canada	1980	NBSS-1: 40–49	NBSS-1:
				Mammography: 2 views then 1 clinical examination every 12 months
			NBSS-2:50-59	S: Self-examination training
				C: Self-examination training + annual history taking
				NBSS-2:
				S: Mammography: 2 views then1 clinical examination every 12 months
				C: Self-examination training and 1 clinical examination every 12 months
Edinburgh [34]	Edinburgh, Scotland	1978	45-64	S: Mammography : 2 views and then 1 view every 24 months
				+ Clinical examination every 12 months
				8 screening rounds
				C: Self-examination
2 Swedish counties [28-30]	Kopparberg, Ostergotland, Sweden	1977 Kopparberg	40-74	Mammography: 1 view
		1978 Ostergoland		- every 24 months for women aged 40 to 49
				-every 33 months for women over 49
				2 screening rounds
New York [31-33]	New York, USA	1963	40-64	Mammography: 2 views and then 1 view every 12 months
				+ Clinical examination every 12 months
				+ History taking only during the 1st round
				4 screening rounds

S: study group; C: control group.

**Figure 2:**
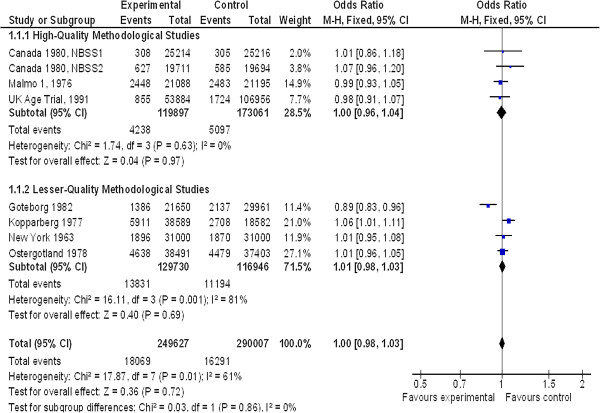
Non-breast cancer mortality in women > 39 years old at 13-year follow-up.

At 13-year follow-up and for all ages (Figure 2), eight out of nine studies were included with a total of 539,634 patients (The Malmo 2 study only had a 9-year follow-up, so it was not included). There was not any significant difference between the two groups, OR = 1.00 (95% CI 0.98 to 1.03) with average heterogeneity I^2^ = 61% (non-breast cancer mortality). For good and moderate methodological quality studies, there was no significant difference between groups: OR = 1.00 (95% CI 0.96 to 1.04) and OR = 1.01 (95% CI 0.98 to 1.03), respectively.

**Figure 3:**
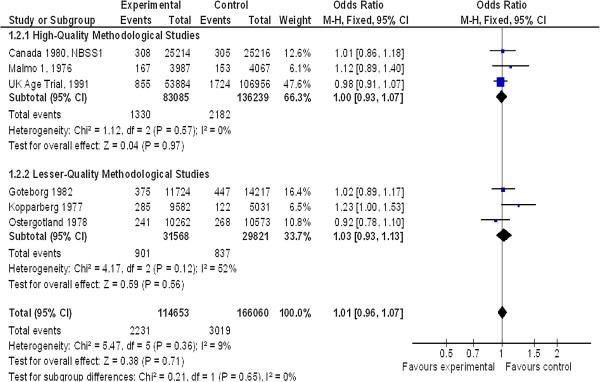
Non-breast cancer mortality in women < 50 years old at 13-year follow-up.

For women under 50 years of age at 13-year follow-up (Figure 3), six studies were included with a total of 280,713 patients. There was no difference between the two groups: OR = 1.01 (95% CI 0.96 to 1.07). For good and moderate methodological quality studies, there was no significant difference between groups: OR = 1.00 (95% CI 0.93 to 1.07) and OR = 1.03 (95% CI 0.93 to 1.13), respectively.

**Figure 4:**
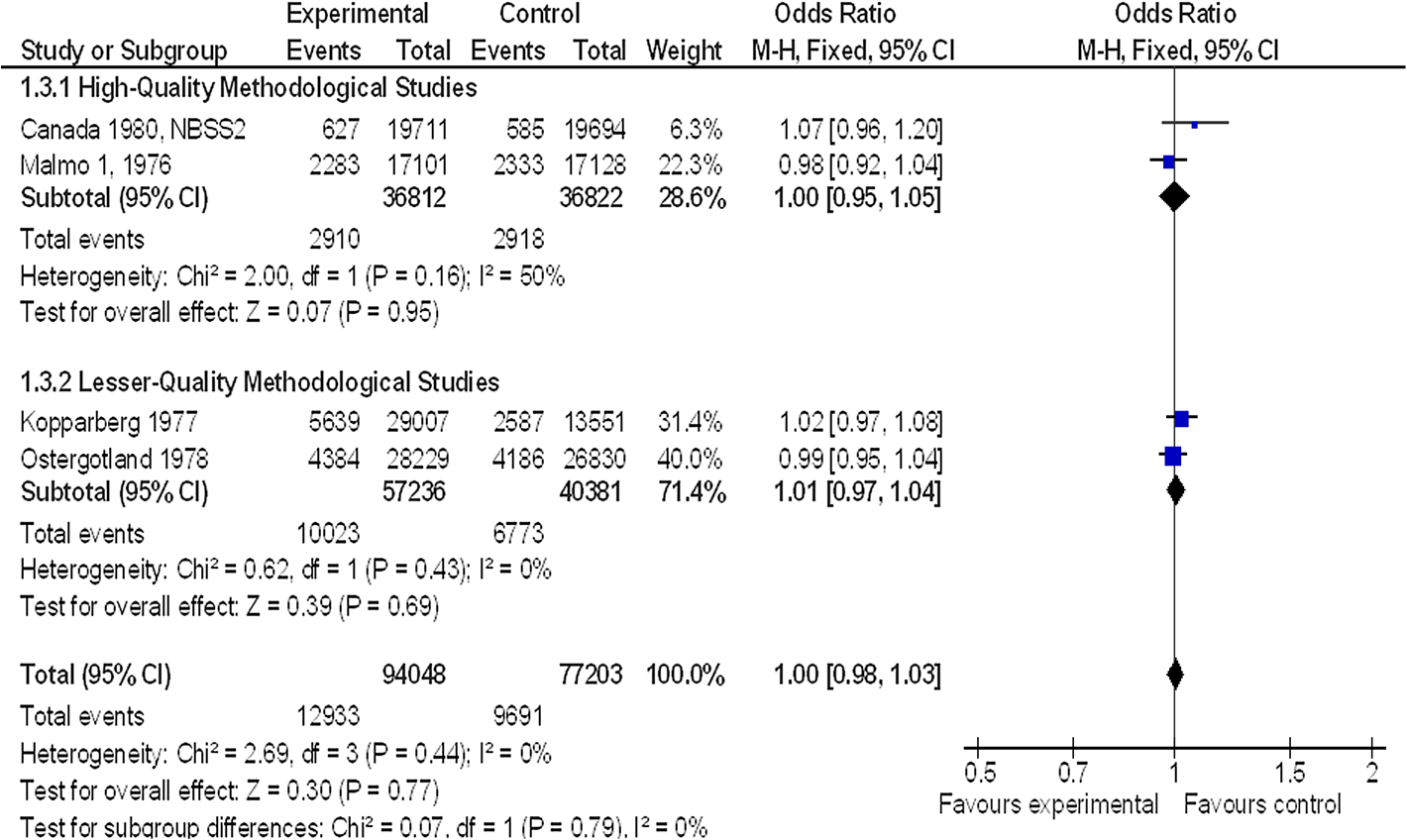
Non-breast cancer mortality in women ≥ 50 years old at 13-year follow-up.

For women over 50 years of age at 13-year follow-up (Figure 4), four studies were included with a total of 22,624 patients. There was no significant difference between groups: OR = 1.00 (95% CI 0.98 to 1.03). The comparison between good and moderate methodological quality studies did not show any difference between groups: OR = 1.00 (95% CI 0.95 to 1.05) and OR = 1.01 (95% CI 0.97 to 1.04), respectively.

The 95% upper CI of the odds ratio is the maximum excess of risk compatible with the data. Based on good-quality methodological studies, this limit was a 3% increase in non-breast cancer mortality, which translated in absolute figures in 12 deaths induced for 100,000 screened women per year.

## Discussion

The lack of change in overall mortality compared with breast cancer mortality reduction in clinical trials on mammography screening led us to wonder about the impact of screening on non-breast cancer mortality. Our meta-analysis is the first one addressing this issue. We showed that in women over 39 years of age at 13-year follow-up, non-breast cancer mortality was unaffected, regardless of the studies’ methodological quality and whether or not patients underwent mammography screening.

This analysis was limited by biases in the included studies (Table 2):

– The drawbacks of the randomization procedure. For example, in the Goteborg trial [24,25], randomization was done by clusters with some time lag between groups. There was a different intervention-to-control ratio in the two age strata (1.2 for women between 39 and 49 years of age, and 1.6 for women between 50 and 59 years of age). Randomization was not appropriately designed because it was based on birth year. In the Malmo2 trial [26,27], an administrative error resulted in a sample size imbalance because all women born in 1934 were recruited in the intervention group. In the same study, recruitment in the screening intervention group was interrupted during some years and repeated two to three times in other years due to a lack of funds.

– The examination types that varied from study to study (time between two mammographies, number of views, combination with breast examination, and mammography quality). The blinded assessment of outcomes assume equal distribution of bias between the compared groups of each study.

– The contamination between groups was 26% in the NBSS1 trial [20].

– The compliance to the screening procedure; for example, limited to 67% in the New York trial [32-34] and 74% in the Malmo1 trial [26,27].

– The outcome classification. In the Malmo1 trial [26], only 73% of death causes were checked through autopsy, with 2% of death causes reclassified and 21% found with multiple cancers. The classification as a breast cancer-related death was interpreted at large, so this may lead to over-diagnosis.

– Our outcome criterion was defined *a posteriori* and supports a *post-hoc* analysis. However, overall and specific mortality are obviously relevant from a clinical point of view, and the discrepancy between the results on these two important outcomes raises questions.

**Table 2:** Bias assessment of included studies

**Clinical trial**	**Randomization**	**Classification of the cause**	**Post-randomization**	**Contamination bias**	**Compliance bias study**
		**of death**	**exclusions**	**control group**	**group**
UK Age Trial [17,18]	Low risk of bias	Low risk of bias	Low risk of bias	No risk of bias	No risk of bias
Goteborg [7,8,19,20]	High risk of bias	Low risk of bias	Low risk of bias	No risk of bias	No risk of bias
Malmö 1 [8,21]	Low risk of bias	Unclear risk of bias	Low risk of bias	Low risk of bias	+
Malmö 2 [7,22]	High risk of bias	Low risk of bias	Unclear risk of bias	No risk of bias	No risk of bias
Stockholm [8,23]	High risk of bias	Unclear risk of bias	Unclear risk of bias	No risk of bias	No risk of bias
Canada 1 [24,27]	Low risk of bias	Low risk of bias	Low risk of bias	+	No risk of bias
Canada 2 [25,26]	Low risk of bias	Low risk of bias	Low risk of bias	+	No risk of bias
2 Swedish counties [28-30]	High risk of bias	High risk of bias	High risk of bias	No risk of bias	No risk of bias
New York [31-33]	High risk of bias	Low risk of bias	Unclear risk of bias	Low risk of bias	+
Edinburgh [34]	High risk of bias	Low risk of bias	High risk of bias	No risk of bias	+

The clinical trials analyzed did not include women with a history of breast cancer. For some of them, recruitment was on a voluntary basis. These facts limit the representativeness of trials in the general population, but do not directly impact the estimate of the intervention effect. The 13-year duration was adopted because it was available in most included studies. A 13-year follow-up includes deaths related to the short- and middle-term consequences of treatments (deaths during surgery and so on) but this length of follow-up may include some long-term mammography-related deaths; for example, deaths related to radio-induced breast cancers. Longer follow-up could have resulted in different results, and it may be interesting to obtain an updated mortality follow-up, but this is beyond the scope of this meta-analysis. The negative effects of screening are well-known and include:

– False positives: for the first round of screening, the rate of false positives was estimated between 4 and 5% [27,36,37]. The recall rate of women after mammography varies between countries. In Norway, Hofvind and colleagues estimated that the cumulative risk of recall was 1 out of 5 (20.8%) during a screening period of 20 years [38]. In the USA, this rate is 49% because of a high rate of prosecutions, the absence of mammography double reading, and the radiologist’s required number of annual mammography readings [39]. In France, this rate is 12% for the initial screening [40]. Recalls have psychological implications: they increase the number of medical visits that may or may not be breast cancer related, and also sadness, anxiety disorders, behavioral disorders, and sexual disorders [41,42].

– Over-diagnosis, and consequently over-treatment: the estimations of over-diagnosis are variable according to the methods used and the adjustments made to take into account these biases. A retrospective Danish study on 57,763 women from 59 to 69 years old having participated from the beginning in the screening campaign organized and followed until 2009 found an over-diagnosis rate of 2.3% [43]. Another retrospective study on 61,568 women from 50 to 69 years old in Florence, Italy, at the beginning of the screening found an over-diagnosis rate of 13% [44]. An Australian modeling study showed that nearly half of all cancers would not have had any clinical impact at 10-year follow-up [45]. In France, the over-diagnosis rate was estimated at 76% for the 50 to 64 years age group (CI 95% 0.67 to 0.85). This was calculated in comparison with similar age cohorts that underwent screening or not (between 1980 and 2005) and in consideration of some exogenous risk factors including obesity, hormone replacement therapy, and alcohol intake [46].

Over-diagnosis and over-treatment are possible explanations for an increase in mortality. It is not known whether the proportion of women unnecessarily treated will die as a result of the treatment. Cancer treatments can cause many adverse effects (risks from surgery, chemotherapy, hormone therapy, and radiation therapy) [47,48]. Even low doses of radiation may cause cancer [49]. The trials supporting this analysis were not useful for exploring these specific hypotheses. Our results suggest that the potential impact of over-diagnosis is not enough to change mortality.

The lack of effect of screening on overall mortality could be explained by a balance between benefit on breast cancer deaths and an increase in other death causes, but also by the inability of these trials to observe significant change on mortality, due to the small proportion of breast cancer deaths (less than 10%) in overall mortality.

The modesty of the benefit size, which was estimated at 1 breast cancer death prevented in 10 years for every 2,000 women screened [12,13], put into question the relevance of mass screening, and highlights the need for clear and complete information for the concerned patients. The benefit-to-risk ratio of screening could be adjusted to the patient profiles following the effect-model approach [50].

## Conclusion

The absence of excess non-breast cancer mortality associated with mammography screening was found in this study. The all-cause death rate was not significantly reduced by screening when compared to the rate observed in unscreened women. Finding more comprehensive or detailed data was difficult given the complexity of studies. Because studies fail to include all aspects of a complex situation, individual modeling could be a solution. These care management models would include all aspects of benefit variation with the best level of evidence on intermediate processes. These models would be validated by comparing them with observational data and clinical trials. This effect model-based approach will help generate individual models of iatrogenic risks and benefits [50]. This will require a regular and independent reassessment of the screening risk/benefit analysis, included in patient education brochures on screening.

## Abbreviations

OR: odds ratio; RCT: randomized controlled trial; RR: relative risk.

## Competing interests

The authors declare that they have no competing interests.

## Authors’ contributions

LF, SE, and FG conceived the study. LF and SE extracted the data and reviewed the selected papers. LF, SE and FG performed statistical analysis. MF, XL, AM, LF, and SE drafted the manuscript. MF, LF, SE, RB, and FG helped interpret the results. All authors read and approved the final manuscript. All authors, external and internal, had full access to all of the data (including statistical reports and tables) in the study and can take responsibility for the integrity of the data and the accuracy of the data analysis.
